# Ionically conducting Li- and Na-phosphonates as organic electrode materials for rechargeable batteries†

**DOI:** 10.1039/d4sc07732f

**Published:** 2024-12-23

**Authors:** Yan Zhang, Petru Apostol, Darsi Rambabu, Xiaolong Guo, Xuelian Liu, Xiaodong Lin, Haijiao Xie, Xiaohua Chen, Koen Robeyns, Jiande Wang, Junzhong Wang, Alexandru Vlad

**Affiliations:** a School of Materials Science and Engineering, Anhui Graphene Carbon Fiber Research Center, Anhui University Hefei 230601 P. R. China wangjz@ahu.edu.cn; b Institute of Condensed Matter and Nanosciences, Molecular Chemistry, Materials and Catalysis, Université Catholique de Louvain Louvain-la-Neuve Belgium jiande.wang@uclouvain.be alexandru.vlad@uclouvain.be; c College of Materials Science and Engineering, Hunan Province Key Laboratory for Advanced Carbon Materials and Applied Technology, Hunan University Changsha 410082 Hunan P. R. China; d Hangzhou Yanqu Information Technology Co., Ltd. P. R. China

## Abstract

Facilitating rapid charge transfer in electrode materials necessitates the optimization of their ionic transport properties. Currently, only a limited number of Li/Na-ion organic cathode materials have been identified, and those exhibiting intrinsic solid-phase ionic conductivity are even rarer. In this study, we present tetra-lithium and sodium salts with the generic formulae: A_4_-Ph-CH_3_P and A_4_-Ph-PhP, wherein A = Li, Na; Ph-CH_3_P = 2,5-dioxido-1,4-phenylene bis(methylphosphinate); Ph-PhP = 2,5-dioxido-1,4-phenylene bis(phenylphosphinate), as novel alkali-ion reservoir cathode materials. Notably, A_4_-Ph-PhP exhibits impressive Li-ion and Na-ion conductivities, measured at 2.6 × 10^−7^ and 1.4 × 10^−7^ S cm^−1^, respectively, in a dry state at 30 °C. To the best of our knowledge, these represent the first example of small-molecule organic cathode materials with intrinsic Li^+^ and Na^+^ conductivity. Theoretical calculations provide further insight into the electrochemical activity of the Li/Na-phenolate groups, as well as the enhanced electron affinity resulting from -phenyl and -Na substitutions. Additionally, Na_4_-Ph-PhP displays two distinct charge–discharge plateaus at approximately 2.2 V and 2.7 V, and 2.0 V and 2.5 V *vs.* Na^+^/Na, respectively, and demonstrates stable cycling performance, with 100 cycles at a rate of 0.1C and an impressive 1000 cycles at 1C. This study not only expands the portfolio of phenolate-based organic salts for use in metal-ion batteries but also underscores the potential of phosphonate-based organic materials in advancing energy storage technologies.

## Abstract 2

Facilitating rapid charge transfer in electrode materials necessitates the optimization of their ionic transport properties.

## Graphical abstract

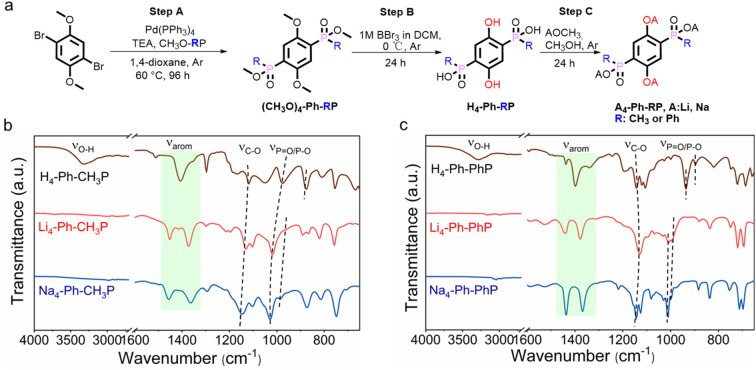

## Introduction

**Scheme 1:**
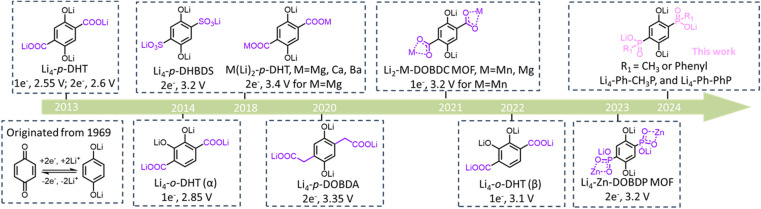
Current Li-phenolate positive materials for organic batteries.

Organic materials have garnered significant attention in the realm of electrochemical energy storage due to their structural flexibility, tunable redox potentials, abundant availability, and cost-effective processing.^1^ Starting from 1969 when Williams *et al.* reported the use of dichloroisocyanuric acid for a 3 V primary Li battery,^2^ a variety of quinone derivatives have been explored as promising positive electrodes in recent years, with most of them, however, prepared and studied in their oxidized state.^3^ Organic alkali-containing materials earned less attention, given also the intrinsic challenges in accessing these, but offer great potential for practical application due to their redox states. Scheme 1 provides a comprehensive summary of lithium-ion positive quinone derivative materials documented in the literature thus far. Poizot and Chen *et al.* examined Li_4_-*p*-DHT (dilithium (2,5-dilithium-oxy)-terephthalate) as a positive material in lithium-ion batteries (LIBs), revealing an average output potential of 2.55 V and 2.6 V *vs.* Li^+^/Li, respectively.^4^ In comparison, dilithium (2,3-dilithium-oxy)-terephthalate, denoted as Li_4_-*o*-DHT (α), demonstrated a higher average potential of 2.85 V *vs.* Li^+^/Li, a 300 mV increase compared to its *para*-position homolog, Li_4_-*p*-DHT.^5^ A notable air-stable lithiated positive material, tetralithium 2,5-dihydroxy-1,4-benzenedisulfonate (Li_4_-*p*-DHBDS), reported by A. E. Lakraychi *et al.*, exhibited a voltage gain of +650 mV higher than that of Li_4_-*p*-DHT.^6^ Poizot *et al.* introduced a new polymorph phase of dilithium (2,3-dilithium-oxy)-terephthalate referred to as Li_4_-*o*-DHT (β), showing a positive potential shift (+250 mV) compared to its α-phase due to crystal arrangement effects.^7^ Subsequently, the same group further developed a cation substitution method using solid carboxy-phenolate salts (Mg(Li_2_)-*p*-DHT), resulting in a remarkable potential increase of 3.4 V *vs.* Li^+^/Li by mitigating donor inductive effects.^8^ Sieuw *et al.* discussed a tetra-lithium 2,5-dihydroxy-1,4-benzenediacetate salt (Li_4_-*p*-DOBDA), which exhibited a two-electron process at an average oxidation potential of 3.35 V *vs.* Li^+^/Li.^9^ Thus, there still is sufficient elbowroom to design new alkali-ion positive quinone-based materials with enhanced electrochemical performance.

Although significant progress has been made in the direction of metal-reservoir organic cathode materials, only a few were reported to be electrically conducting, whereas none were reported to be ionically conductive. The electrical and ionic conductivities of organic electrode materials are critical parameters that directly influence the kinetics of electrode reactions, thereby playing a pivotal role in determining the overall electrochemical performance. Recent studies on ionic conducting OMs have primarily focused on electrolytes, including commercial liquid and novel solid electrolytes, resulting in notable advancements.^10^ Nevertheless, the transport of alkali metal ions in organic electrode materials (OEMs) poses a limiting factor for overall ion transport. Therefore, it is crucial to explore novel organic electrode materials (OEMs) with improved ionic conductivity to achieve well-defined alkali metal ion transfer kinetics.

Being aware of this limitation, a significant advancement was made by Darsi *et al.*, who reported a quinone-based electrode material, Li_2_-Mn-DOBDC, as the first redox-active Li^+^ reservoir metal–organic framework (MOF), which displayed an average discharge potential of 3.2 V *vs.* Li^+^/Li and an electrical conductivity of 10^−7^ S cm^−1^ at room temperature, showcasing its potential for improving the performance of organic electrode materials.^11^ However, Li_2_-Mn-DOBDC was found to be a non-conductor in the solid phase, owing to strong electrostatic interaction of Li^+^ with the phenoxide group. Recently, the Li_4_-Zn-DOBDP MOF was reported as a pioneering example of a non-solvated cation-conducting MOF, demonstrating an average discharge potential of 3.2 V *vs.* Li^+^/Li and fast Li-ion transfer kinetics.^12^ The development of Li-reservoir ionically conductive cathode materials with redox-active organic moieties is still in its infancy. Apart from the reported MOF composition, no small molecule organic cathode has yet been demonstrated to facilitate Li-ion conduction pathways, highlighting a significant gap in the field and an opportunity for further exploration. Developing and understanding new organic Li-reservoir cathode materials remains thus an important area of research.

**Fig. 1:**
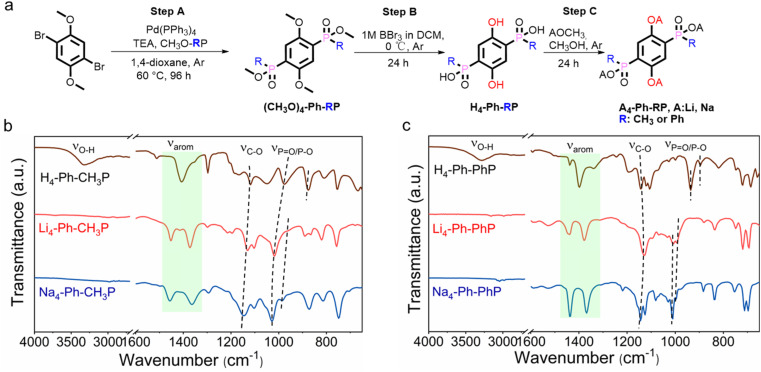
(a) Synthetic pathway for A_4_-Ph-RP, A = Li or Na and R = CH_3_ or Ph. Comparative FTIR analysis of the methyl version (b) and the phenyl version (c) and their corresponding lithium and sodium salts.

Li_6_-DOBDP (fully lithiated 2,5-dihydroxy-1,4-benzenediphosphonic acid, or H_6_-DOBDP) is regarded as another promising candidate for Li-reservoir cathode materials, attributed to the relatively high electron-withdrawing effect of its phosphonate groups. However, H_6_-DOBDP contains only two characteristic acidic protons from the phosphonate hydroxy groups, with p*K*_a_ values ranging from 1.1 to 2.3 for p*K*_a1_ and 5.3 to 7.2 for p*K*_a2_, respectively.^13^ The complete lithiation of H_6_-DOBDP *via* an acid–base reaction was unsuccessful due to the higher p*K*_a_ values of the H_6−*x*_Li_*x*_-DOBDP (4 < *x*< 6) protons. Herein, to address the challenging lithiation of H_6_-DOBDP, we delicately designed and successfully synthesized H_4_-Ph-CH_3_P (phenylene-1,4-bis(methylphosphinic acid)) and H_4_-Ph-PhP (phenylene-1,4-bis(phenylphosphinic acid)) by replacing one –OH group on each of the phosphonic acid groups with methyl and phenyl groups, respectively (Fig. 1a). Through theoretical calculations, we provide a comprehensive understanding of the influence of substituents and various salts on these series of novel materials and illustrate that Li/Na-phenolate serve as redox active centers. In addition, both Li_4_-Ph-PhP and Na_4_-Ph-PhP show an ionic conductivity (Li^+^ and Na^+^) of approximately 10^−7^ S cm^−1^ in the dry phase, which is the first report of redox active small organic electrode materials displaying ionic conductivity. Another significant result arising from the incorporation of the phenyl-phosphonate group is the substantial elevation of redox potential, exhibiting a remarkable increase of +550 mV when compared to Li_4_-*p*-DHT^4*a*^ (2.55 V *vs.* Li^+^/Li). Furthermore, Na_4_-Ph-PhP, as a Na-reservoir cathode material, shows stable cycling over 100 cycles at 0.1C (1C = 112 mA g^−1^) with a capacity retention of 73%, and the battery also shows excellent rate capability, being able to sustain a high rate of 10C.

## Results and discussion

Li_6_-DOBDP serves as a fundamental structural unit featuring phosphonate functionality (a strong electron withdrawing group), similar to Li_4_-*p*-DHT^4*a*^ and Li_4_-*p*-DHBDS.^6^ Nonetheless, attempts to synthesize Li_6_-DOBDP through conventional acid–base reactions were unsuccessful due to the elevated p*K*_a_ of H_6−*x*_Li_*x*_-DOBDP (*x* < 6), a phenomenon discernible from the retained characteristic –OH group depicted in Fig. S1.† These efforts led us to modify hexa-anionic DOBDP^6−^ to a tetra-anionic form by replacing one –OH on each phosphonate with a methyl or phenyl group. This approach is expected to augment the ligand's acidity, thereby facilitating quantitative lithiation and sodiation, as observed in A_4_-Ph-RP (R = CH_3_, Ph).

The synthesis procedure of organo-phospho-phenolate compounds with methyl or phenyl substituents is schematically presented in Fig. 1a and the ESI.† Steps A and B are based on the substitution reaction outlined in the reported protocols, with specific adjustments made for this particular case.^14^ The analogues H_4_-Ph-RP (where R = CH_3_ or Ph) were synthesized following a demethylation method (step B), which involved the use of an excess of boron tribromide (BBr_3_) to ensure the complete conversion of –OCH_3_ into –OH. In step C, the complete lithiation/sodiation of H_4_-Ph-RP was accomplished by reacting it with metal methoxide (AOCH_3_) in methanol (A = Li or Na), resulting in four organo-phospho-phenolate alkali salts.

The protonated versions and synthesis intermediates in this work were characterized and confirmed by ^1^H-NMR spectroscopy and HRMS (Table S1 and Fig. S2–S9†). Subsequently, quantitative lithiation/sodiation of H_4_-Ph-RP (R = CH_3_ or Ph) was confirmed by FTIR analysis. As shown in Fig. 1b and c, the disappearance of the broad phosphate and phenolic –OH band at around 3200 cm^−1^, along with a slight shift in the C=C and P–O/P=O bands in Li_4_-Ph-PhP and Na_4_-Ph-PhP, was observed, confirming successful lithiation/sodiation.^15^ Powder XRD analysis revealed that Na_4_-Ph-PhP and Li_4_-Ph-CH_3_P are crystalline, while Li_4_-Ph-PhP and Na_4_-Ph-CH_3_P occur as amorphous phases. Additionally, Li_4_-Ph-PhP and Na_4_-Ph-PhP present similar PXRD patterns (Fig. S10†). A comparison with the protonated H_4_-Ph-RP (R = CH_3_ or Ph) revealed that the diffraction peaks of the corresponding salts were shifted to lower 2theta angles. This shift suggests an alteration in the unit cell and structural arrangement, providing additional evidence for the formation of a framework with Li/Na.^1*l*,*m*^

**Fig. 2:**
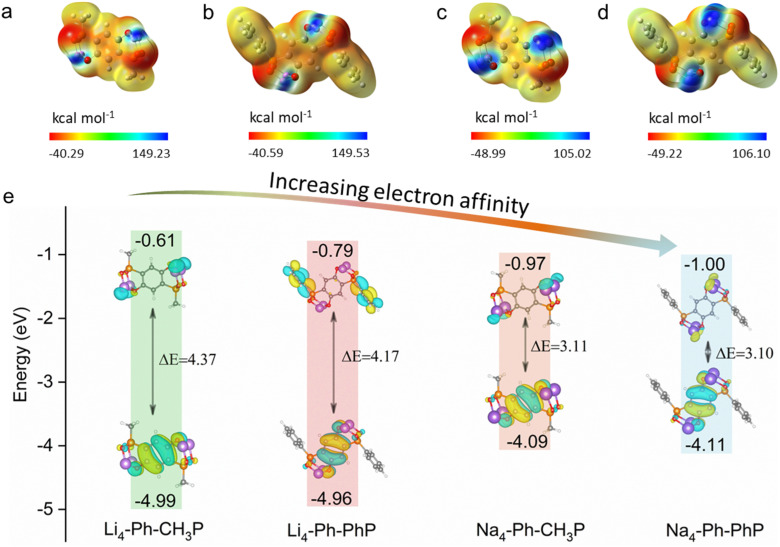
Calculated ESP maps of (a) Li_4_-Ph-CH_3_P, (b) Li_4_-Ph-PhP, (c) Na_4_-Ph-CH_3_P and (d) Na_4_-Ph-PhP, (e) HOMO–LUMO energy level diagram.

As novel Li/Na-containing organic positive electrodes, the molecular electrostatic potential (MESP) approach of Li_4_-Ph-CH_3_P, Li_4_-Ph-PhP, Na_4_-Ph-CH_3_P and Na_4_-Ph-PhP was employed to deduce the potential active sites for Li-ion and Na-ion storage (Fig. 2a–d). Regions with higher positive ESP are more likely to participate in nucleophilic reactions, while regions with more negative ESP favor electrophilic reactions.^16^ The initial charging process involves the extraction of Li/Na-ions and the loss of electrons. The nucleophilic centers (highlighted by blue regions) are identified as highly reactive sites. The ESP mapping results reveal that regions near the Li/Na-enolate exhibit higher positive ESP values. Thus, phenolates (C-OLi and C-ONa) are identified as the active sites. Furthermore, density functional theory (DFT) calculations were conducted to examine the detailed impact of substituent groups (-CH_3_/-phenyl) and salts (-Li/-Na) on the geometries and frontier molecular orbitals of a series of phospho-phenolate organic materials, including the lowest unoccupied molecular orbital (LUMO) and the highest occupied molecular orbital (HOMO). As depicted in Fig. 2e, the LUMO energy level decreases slightly from −0.61 eV in Li_4_-Ph-CH_3_P to −0.79 eV in Li_4_-Ph-PhP, which is also observed in Na_4_-Ph-CH_3_P (−0.97 eV) and Na_4_-Ph-PhP (−1.00 eV), caused by strong electron-withdrawing capabilities of -phenyl. In addition, the LUMO is also affected by the presence of different salts (-Li/-Na) in methyl and phenyl versions. Obviously, Na_4_-Ph-CH_3_P and Na_4_-Ph-PhP exhibit much lower LUMO energy levels when compared to Li_4_-Ph-CH_3_P and Li_4_-Ph-PhP, given the lower polarizing power of Na^+^.^1*g*,*m*,17^ The energy gaps between the HOMO and LUMO of organic materials are a critical factor used to assess their intrinsic electronic conductivity.^18^ The phenyl version shows a much lower energy gap (4.17 eV for Li_4_-Ph-PhP and 3.10 eV for Na_4_-Ph-PhP), which is beneficial for quick Li/Na-ion storage (Fig. 2e and S12†). Therefore, Li_4_-Ph-PhP and Na_4_-Ph-PhP can serve as potential organic positive electrode materials with good electron conductivity and electrochemical charge–discharge capabilities.

**Fig. 3:**
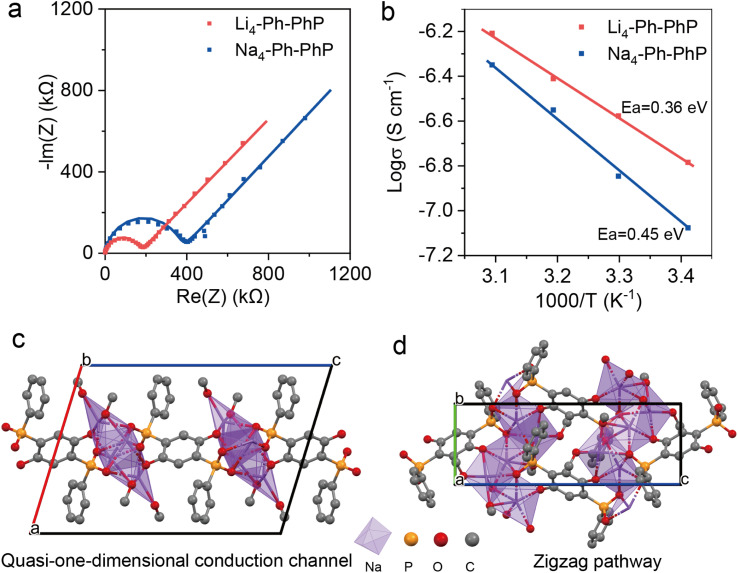
(a) Nyquist plots for Li_4_-Ph-PhP and Na_4_-Ph-PhP at 30 °C, (b) Arrhenius plot of ionic conductivity of A_4_-Ph-PhP (A = Li, Na), (c and d) structural representation of Na_4_-Ph-PhP highlighting the one-dimensional Na chain along the *b* axis.

The ionic conductivity of electrode materials is crucial in determining the performance of energy storage devices. Ionically conducting OEMs are particularly advantageous, as sluggish mobility of Li/Na ions within the bulk of the electrodes can result in reduced rate capability. The ionic conductivity of A_4_-Ph-PhP (A = Li, Na) was investigated through variable-temperature measurements using alternating current (a.c.) impedance spectroscopy of powder formed pellets. Nyquist plots exhibit a semicircle in the high frequency region, and a linear tail in the low-frequency range, with this feature being assigned to the ionic response of the system to the external a.c. field.^10*a*,19^ The resistance of different samples was calculated by fitting with an equivalent circuit with values of approximately ∼10^−7^ S cm^−1^ in the temperature range of 20 to 50 °C (Fig. 3b, S13 and Table S2†). Fitting the temperature dependent conductivity to the linearized Nernst–Einstein relation yielded activation energies of 0.36 and 0.45 eV for Li_4_-Ph-PhP and Na_4_-Ph-PhP, respectively. It is worth mentioning that the Li-ion ionic conductivity is higher than that of the Na-ion (*σ*(Li^+^) > *σ*(Na^+^)), attributed to the smaller ionic radius of Li^+^ which facilitates ion mobility^1*m*,20^. According to the PXRD data, Li_4_-Ph-PhP and Na_4_-Ph-PhP exhibit comparable patterns, however, PXRD peaks of Li_4_-Ph-PhP are drastically diminished (Fig. S10†). To better understand the ionic conductivity of the materials, we solved the crystal structure of Na_4_-Ph-PhP. The 2D layered structure of Na_4_-Ph-PhP was determined by single crystal X-ray diffraction. Na_4_-Ph-PhP crystallizes in the monoclinic space group *P*2_1_/*c*, where one sodium atom is coordinated in a disordered trigonal bipyramidal geometry by oxygen atoms: three bonds are formed with oxygen atoms from phosphonate groups (O–P), one bond with a phenolic oxygen atom, and one bond with an oxygen atom from methanol. The second sodium atom is coordinated to two oxygen atoms from methanol, one phenolic oxygen atom, and two oxygen atoms from phosphonate groups (O–P). This coordinated sodium helps interconnect three bridging phosphonate linkers, forming two-dimensional layers along the *b*-axis, with a distance of 12.66 Å between them along the *a*-axis. The coordinated methanol molecules are positioned between these layers (Fig. 3c, d, S14 and Table S3†). After desolvation of the structure, the material remain highly crystalline, with only a shift of the main peak to a higher angle and those powder pattern match very well with the one from the bulk synthesis of Na_4_-Ph-PhP (Fig. S15†). From these structural details, it is worthy to assume that the sodium is bonded strongly to the linkers, and its movement is predominantly restricted to a single dimension (Fig. 3d). This unique pathway can significantly decrease the hopping distance and thus impact the material's ionic conductivity.

**Fig. 4:**
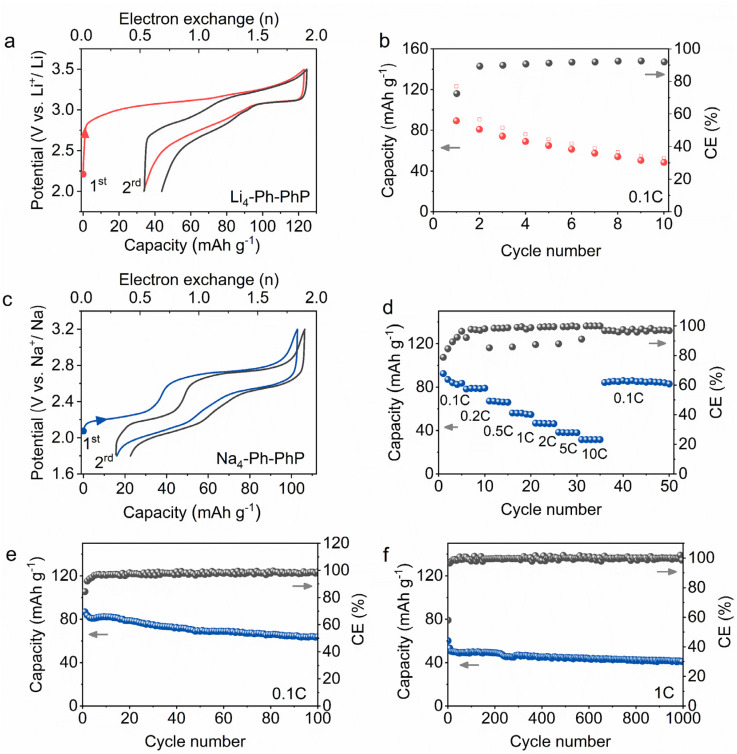
Electrochemical performance of Li_4_-Ph-PhP and Na_4_-Ph-PhP as positive electrode materials for charge storage. Li_4_-Ph-PhP: (a) the first two charge and discharge voltage profiles at 0.1C and (b) cycling performance at 0.1C; Na_4_-Ph-PhP: (c) the first two charge and discharge voltage profiles at 0.1C, (d) capacity-rate from 0.1C to 10C per series of five cycles, and cycling performance at (e) 0.1C and (f) 1C.

The electrochemical performances of Li_4_/Na_4_-Ph-CH_3_P and Li_4_/Na_4_-Ph-PhP in half cells are displayed in Fig. 4 and S16.† Both Li_4_-Ph-CH_3_P and Na_4_-Ph-CH_3_P show low first cycle charge and discharge capacity, and we attribute the low capacity and reversibility to either the high instability of their one-electron oxidized radical state or the higher solubility of active materials in comparison to their -PhP counterparts (Fig. S16and S17†). In contrast, Li_4_/Na_4_-Ph-PhP were found to exhibit notably enhanced battery performances compared to Li_4_/Na_4_-Ph-CH_3_P homologues (Fig. 4 and S16†). The additional two phenyl rings attached to the P atom could effectively stabilize the formed radical cation at the half-oxidized state. As shown in Fig. 4a, the first oxidation of Li_4_-Ph-PhP is characterized by an average plateau at 3.1 V *vs.* Li^+^/Li, corresponding to the exchange of approximately 1.9 Li^+^ equivalents per formula unit and yielding a charge-specific capacity of 122 mA h g^−1^. This value is close to the theoretical lithium storage capacity of 2 Li^+^ equivalents, or *Q*_theo._ = 129 mA h g^−1^. This indicates that the reduced form of Li_4_-Ph-PhP has low solubility in the electrolyte, and nearly all the electroactive material can be electrochemically accessed. Nevertheless, capacity fade is observed in the subsequent cycles, indicating the partial solubility of Li_4_-Ph-PhP in the electrolyte. After 10 cycles, the reversible charge and discharge capacities of Li_4_-Ph-PhP are 52 mA h g^−1^ and 48 mA h g^−1^, respectively, with a coulombic efficiency (CE) of approximately 92.3% (Fig. 4b).

Na_4_-Ph-PhP exhibits an efficient two-electron reversible charging–discharging profile, with two plateaus located at approximately 2.2 V and 2.7 V during charging and 2.0 V and 2.5 V *vs.* Na^+^/Na during discharging (Fig. 4c). During the first charge cycle, approximately 1.8 Na^+^ ions are extracted per unit of Na_4_-Ph-PhP, yielding a specific charge capacity of 102 mA h g^−1^, closely approaching its theoretical capacity (*Q*_theo._ = 112 mA h g^−1^), suggesting the low solubility of the reduced state of Na_4_-Ph-PhP in the electrolyte. In the discharge process, the discharge capacity of Na_4_-Ph-PhP is 87 mA h g^−1^, with a first coulombic efficiency of 84.3%. In the second cycle of charge–discharge, the charge specific capacity and discharge specific capacity are 91 mA h g^−1^ and 84 mA h g^−1^, respectively, with a coulombic efficiency of 92.3%, which indicated the lower solubility of the oxidized state during charge discharge cycles. The rate capability was investigated at different cycling rates ranging from 0.1C to 10C (Fig. 4d). At current densities of 0.1C, 0.2C, 0.5C, 1C, 2C, 5C, and 10C, the reversible discharge capacities of Na_4_-Ph-PhP are 82 mA h g^−1^, 78 mA h g^−1^, 66 mA h g^−1^, 54 mA h g^−1^, 45 mA h g^−1^, 38 mA h g^−1^, and 31 mA h g^−1^, respectively. When the current is restored to the initial current density of 0.1C, the reversible discharge capacity recovers to 81 mA h g^−1^, demonstrating good reversibility and cycle stability. At a low current density of 0.1C, Na_4_-Ph-PhP exhibits a reversible charge–discharge capacity of 64 mA h g^−1^ and 63 mA h g^−1^ after 100 cycles (nearly three months of testing), with a coulombic efficiency of 98.4% (Fig. 4e). Even at a high current density of 1C, Na_4_-Ph-PhP still maintains a reversible capacity of 41 mA h g^−1^ after 1000 cycles, with a coulombic efficiency of 99.3% (Fig. 4f). Therefore, the ionic conductivity and reversible redox activity on carbonyl/quinone groups (Fig. S18†) of Na_4_-Ph-PhP contribute to its excellent cycling stability and rate performance. However, further studies are needed to tackle the solubility issue of this class of material and eventually improve the cycling and rate capability of these new novel phosphonate organic positive materials by optimizing their structures and incorporating additional functionalities.

## Conclusions

We present a novel series of phenylphosphate-based carbonyl compounds as new organic positive materials, assessing their ionic conduction and charge storage properties. The ESP and LUMO–HOMO calculation methods reveal the electrochemical activity of Li/Na-phenolate groups, providing a comprehensive evaluation of the enhanced electron affinity induced by the -phenyl and -Na substitutions. Furthermore, both Li-ion and Na-ion phases demonstrate ionic conductivities of approximately 10^−7^ S cm^−1^, establishing this class of materials as the first redox-active organic cathode with reported intrinsic ionic conductivity. A systematic investigation is carried out to elucidate the structure–performance relationship of Na_4_-Ph-PhP. During the charge/discharge process, Na_4_-Ph-PhP was found to undergo a reversible two-electron process with good cycling stability and rate performances. This study not only unveils new Li/Na-containing organic positive electrode materials but also establishes a promising pathway for the development of organic phosphonate-based materials with promising ionic conductivity for future practical applications.

## Data availability

The data that support the findings of this study are available from the corresponding author upon reasonable request.

## Author contributions

J. D. W. and A. V. conceived of the presented idea. Y. Z. and P. A. contributed equally to this work. Y. Z and P. A. carried out the experiments, interpreted the experimental data and wrote the manuscript. J. D. W. helped analyze the data. D. R., X. L. G., X. L. L. X. D. L., X. H. C. helped analyze and revise the manuscript. H. J. X. performed the calculations. K. R. performed a single crystal test and analyzed the structure. A. V. and J. Z. W. supervised the funding of this work. All authors discussed the results and contributed to the final manuscript.

## Conflicts of interest

The authors declare no conflicts of interest.

## Supplementary Material

SC-OLF-D4SC07732F-s001

SC-OLF-D4SC07732F-s002
