# Supplementary material for: Ionically conducting Li- and Na-phosphonates as organic electrode materials for rechargeable batteries
Source: Chem Sci. 2024 Dec 23;16(4):1819–25. doi: 10.1039/d4sc07732f (PMC11664480; doi:10.1039/d4sc07732f)
Supplement: SC-016-D4SC07732F-s001 [file SC-016-D4SC07732F-s001.pdf]

Supplementary Information (SI) for Chemical Science.
This journal is © The Royal Society of Chemistry 2024

Supporting Information:

Ionically Conducting Li- and Na-Phosphonates as Organic Electrode Materials for Rechargeable Batteries

Yan Zhang[§,a,b,c], Petru Apostol[§,b], Darsi Rambabu[b], Xiaolong Guo[b], Xuelian Liu[b], Xiaodong Lin[b], Haijiao Xie[d], Xiaohua Chen[c], Koen Robeyns[b], Jiande Wang[b,*], Junzhong Wang[a,*], Alexandru Vlad [b,*]

[a] *School of Materials Science and Engineering, Anhui Graphene Carbon Fiber Research Center, Anhui University, Hefei, 230601 P. R. China.*

[b] *Institute of Condensed Matter and Nanosciences, Molecular Chemistry, Materials and Catalysis, Université catholique de Louvain, Louvain-la-Neuve, Belgium.*

[c] *College of Materials Science and Engineering, Hunan Province Key Laboratory for Advanced Carbon Materials and Applied Technology, Hunan University, Changsha 410082, Hunan, P. R. China.*

[d] *Hangzhou Yanqu Information Technology Co., Ltd.*

[§] *These authors contributed equally to this work.*

Table of Contents

Materials and methods 4

1.1 Reagents and chemicals 4

1.2 Synthesis procedure 4

1.2.1 Synthesis of methyl R-phosphinate (CH_3O-CH_3P and CH_3O-PhP) 4

1.2.2 Syntheses of Dimethyl (2,5-dimethoxy-1,4-phenylene) bis(R-phosphinate) (($(CH_3O)_4$-Ph-CH_3P and $(CH_3O)_4$-Ph-PhP) 4

1.2.3 Synthesis of H_4-Ph-CH_3P and H_4-Ph-PhP 5

1.2.4 Synthesis of $H_{6-x}Li_x$-DOBDP ($0<x<6$), Li_4-Ph-CH_3P and Li_4-Ph-PhP 5

1.2.5 Synthesis of Na_4-Ph-CH_3P and Na_4-Ph-PhP 5

1.2.6 Synthesis of Na_4-Ph-PhP crystals 5

1.3 Materials characterizations 6

1.4 Ionic conductivity test 6

1.5 Electrochemical cell assembly and analysis 6

1.6 Density Functional Theory Simulation 7

Figure S1. (a) Comparative FTIR analysis of H_6-DOBDP and $H_{6-x}Li_x$-DOBDP, (b) Schematic illustration of lithiation reaction of H_6-DOBDP by conventional liquid acid-base reaction conditions. 8

Table S1. List of the studied compounds with the full names and abbreviations. 9

Figure S2. ^1H NMR of CH_3O-CH_3P in $CDCl_3$. 10

Figure S3. ^1H NMR of $(CH_3O)_4$-Ph-CH_3P in $CDCl_3$. 11

Figure S4. ^1H NMR of H_4-Ph-CH_3P in CD_3OD. 12

Figure S5. ^1H NMR of CH_3O-PhP in $CDCl_3$. 13

Figure S6. ^1H NMR of $(CH_3O)_4$-Ph-PhP in $CDCl_3$. 14

Figure S7. ^1H NMR of H_4-Ph-PhP in DMSO-d_6. 15

Figure S8. HR-MS of H_4-Ph-CH_3P. 16

Figure S9. HR-MS of H_4-Ph-PhP. 17

Figure S10. PXRD patterns of synthesized materials. 18

Figure S11. TG of (a) A_4-Ph-CH_3P, and (b) A_4-Ph-PhP, A=Li, Na. 19

Figure S12. Density of state (DOS) of Li_4-Ph-CH_3P, Na_4-Ph-CH_3P, Li_4-Ph-PhP, and Na_4-Ph-PhP. 20

Figure S13. Nyquist plots of (a) Li_4-Ph-PhP and (b) Na_4-Ph-PhP from 20 °C to 50 °C. 21

Table S2. Ionic conductivity calculation details for Li_4-Ph-PhP and Na_4-Ph-PhP, pellet area A=0.785 cm^2, L is the thickness of the pellets. 22

Figure S14. X-ray crystal structure of Na_4-Ph-PhP·CH_3OH. 23

Figure S15. PXRD patterns of Na_4-Ph-PhP materials. 25

Figure S16. The first charge and discharge voltage profiles at a rate of 0.1C for (a) Li_4-Ph-CH_3P, (b) Na_4-Ph-CH_3P electrode materials. 26

Figure S17. Photographs of glass fiber separator from (a) Li_4-Ph-CH_3P and (b) Na_4-Ph-CH_3P cells after the first charge/discharge process. 27

Figure S18 (a) Ex-situ FTIR spectroscopy analysis of Na_4-Ph-PhP in different charge and discharge state, (b) redox mechanism of A_4-Ph-RP. 28

Materials and methods

1.1 Reagents and chemicals

Benzene, 1,4-Dibromo-2,5-dimethoxybenzene, tetrakis (triphenylphosphine) palladium (0) (Pd $(PPh_3)_4$) and 1,4-dioxane were purchased from TCI chemicals. Dichlorophenylphosphine ($PhPCl_2$), methyldichlorophosphane (CH_3PCl_2) and dichloromethane (DCM) were purchased from FisherSci. Boron tribromide (BBr_3, 1 M soln. in dichloromethane) and anhydrous methanol (CH_3OH) were purchased from Alfa Aesar. Sodium sulphate anhydrous and diethyl ether were purchased from VWR Chemicals. Triethylamine (TEA) was purchased from Acros Organics. Lithium methoxide ($LiOCH_3$) and sodium methoxide ($NaOCH_3$) were purchased from Sigma Aldrich. 2,5-Dihydroxy-1,4-benzenediphosphonic acid (H_6-DOBDP) was obtained by from Epsilon Chimie. All chemicals were used as received.

1.2 Synthesis procedure

1.2.1 Synthesis of methyl R-phosphinate (CH_3O-CH_3P and CH_3O-PhP)

A first flask was charged with $RPCl_2$ (R=CH_3 or Ph), three times evacuated and flushed with argon (Ar), and diluted with dry benzene (8 mL per 1 g of $RPCl_2$). In a second flask, 2.4 molar equivalents of dry methanol were mixed with 1 molar equivalent of dry triethylamine under Ar atmosphere. The mixture was cooled in an ice bath, and the solution of $RPCl_2$ was added slowly. The formed precipitate was filtered and the filtrate was evaporated to form an oily product which was directly used without further purification.

CH_3O-CH_3P: Yield: 43 %. ^{1}H NMR (300 MHz, $CDCl_3$) δ 7.97 (q, J = 2.1 Hz, 0.5H), 6.17 (q, J = 2.1 Hz, 0.5H), 3.68 (d, J = 12.1 Hz, 3H), 1.43 (ddd, J = 15.1, 8.9, 2.1 Hz, 3H).

CH_3O-PhP: Yield: 75 %. ^{1}H NMR (300 MHz, $CDCl_3$) δ 8.41 (s, 0.5H), 7.75-7.66 (m, 2H), 7.56-7.48 (m, 1H), 7.47-7.38 (m, 2H), 6.53 (s, 0.5H), 3.72-3.66 (m, 3H).

1.2.2 Syntheses of Dimethyl (2,5-dimethoxy-1,4-phenylene) bis(R-phosphinate) ($(CH_3O)_4$-Ph-CH_3P and $(CH_3O)_4$-Ph-PhP)

A mixture of 3.55 g of 1,4-Dibromo-2,5-dimethoxybenzene (12 mmol) and 1 g of $Pd(PPh_3)_4$ (0.86 mmol) was added into a flask under an Ar atmosphere. Then, 125 mL dry 1,4-dioxane was added, followed by 4.0 mL dry triethylamine (28 mmol) and CH_3O-CH_3P (36 mmol) (or CH_3O-PhP (36 mmol)). The mixture was stirred at 60 °C for 96 hours. After cooling to room temperature, the formed precipitate was filtered off and the filtrate was evaporated to dryness. The solid residue was dissolved in DCM and washed with water 3 times. The purification process was individual for each product, as seen below.

$(CH_3O)_4$-Ph-CH_3P: The DCM phase was evaporated to dryness to obtain a light-yellow powder with high purity. Yield: 27%. ^{1}H NMR (300 MHz, $CDCl_3$) δ 7.66-7.52 (m, 2H), 3.99-3.79 (m, 6H), 3.67-3.50 (m, 6H), 1.77 (ddd, J = 15.6, 5.7, 3.8 Hz, 6H).

$(CH_3O)_4$-Ph-PhP: The organic phase was dried over sodium sulfate (anhydrous). The solution was evaporated, and the product was precipitated by adding diethyl ether, then filtered off and dried at 80 °C to get $(CH_3O)_4$-Ph-PhP as a light-yellow powder. Yield: 66%. ^{1}H NMR (300 MHz, $CDCl_3$) δ 7.95-7.76 (m, 4H), 7.61-7.55 (m, 2H), 7.52-7.48 (m, 2H), 7.47-7.38 (m, 4H), 3.83-3.66 (m, 12H).

1.2.3 Synthesis of H_4-Ph-CH_3P and H_4-Ph-PhP

To a solution of $(CH_3O)_4$-Ph-CH_3P (or $(CH_3O)_4$-Ph-PhP) in DCM under Ar atmosphere, 7 molar equivalents of BBr_3 (1 M in DCM) was added dropwise at 0 °C. After 24 hours, DI-water (20 ml) was added to quench the reaction, then filtered off and washed with DCM, and dried at 80°C.

H_4-Ph-CH_3P: ^{1}H NMR (300 MHz, MeOD) δ 7.16 (dd, J = 14.0, 6.5 Hz, 2H), 1.75 (dd, J = 19.6, 16.1 Hz, 6H).

H_4-Ph-PhP: ^{1}H NMR (300 MHz, DMSO) δ 10.47 (s, 4H), 7.81-7.68 (m, 4H), 7.54 (ddt, J = 6.0, 2.9, 1.4 Hz, 2H), 7.51-7.41 (m, 4H), 7.22-6.77 (m, 2H).

1.2.4 Synthesis of $H_{6-x}Li_x$-DOBDP ($0<x<6$), Li_4-Ph-CH_3P and Li_4-Ph-PhP

The lithiation was performed in anhydrous methanol with a stoichiometric amount of lithium methoxide. The mixture was stirred at room temperature inside an Ar-filled glovebox for 24 hours, and then added diethyl ether to wash it for three times. After filtration, $H_{6-x}Li_x$-DOBDP, Li_4-Ph-CH_3P and Li_4-Ph-PhP were transferred to a BUCHI glass oven to remove methanol and diethyl ether at 140 °C, 150 °C and 220 °C, respectively.

1.2.5 Synthesis of Na_4-Ph-CH_3P and Na_4-Ph-PhP

The sodiation was carried out in anhydrous methanol with a stoichiometric amount of sodium methoxide. This mixture was stirred at room temperature for 24 hours inside an Ar-filled glovebox, and washed with diethyl ether. After filtered, Na_4-Ph-CH_3P and Na_4-Ph-PhP were transferred to a BUCHI glass oven to remove methanol and diethyl ether at 150 °C and 220 °C, respectively.

1.2.6 Synthesis of Na_4-Ph-PhP crystals

Needle-shaped crystals of Na_4-Ph-PhP, suitable for X-ray analysis, were obtained by slow diffusion of diethyl ether into a methanolic solution of Na_4-Ph-PhP (1.5 mg mL^{-1}) at room temperature in an argon filled glove box over three days.

1.3 Materials characterizations

Powder X-ray diffraction (PXRD) was acquired using a STOE Stadi P diffractometer equipped with monochromated Mo Kα radiation. Patterns were collected in the 2θ range of 2-35°. FTIR spectroscopy was carried out on pristine powders using an Agilent Technologies Cary 630 spectrometer with a Single reflection ATR module. Thermogravimetric analysis (TGA) was carried out under argon with a SENSYSevo instrument from Setaram at a heating rate of 10 °C min^{-1} between 25 and 800 °C.

X-ray crystallography

Single crystal X-ray data collection on Na4-Ph-PhP was carried out on a MAR345 image plate detector using Mo-Kα radiation (0.71073 Å) generated by an Incoatec IμS microfocus source (Montel mirrors). Data integration and reduction were performed by CrysAlis^{PRO}[1] and the implemented absorption correction was applied. Beyond 0.93 Å the crystal diffracted poorly and a data cut-off was imposed during integration. The structure solution was performed by dual-space direct methods in SHELXT[2] and the structure was further refined against F^2 using SHELXL2019/3[3]. Non-hydrogen atoms were refined anisotropically; H_{CH} atoms were added in calculated positions; H_{OH} atoms were either located from a Fourier map or added in calculated positions and refined in riding mode. Symmetry analysis and validation were carried out using PLATON.[4] Figures were made using the molecular visualization software Mercury v4.3.[5] CCDC 2401944 includes the supplementary crystallographic data and can be downloaded free of charge from The Cambridge Crystallographic Data Centre via www.ccdc.cam.ac.uk/structures.

1.4 Ionic conductivity test

Pellets (10 mm in diameter) were prepared by cold pressing the powders at an applied pressure of 5.0 T for 2 min under a protective Ar atmosphere. The powder was pressed between two carbon coated Aluminum foils to insure a good contact. In Ar-filled glovebox, the pellets were assembled in home-made Swagelok cells, and transferred to a climatic chamber for variable temperature tests. Measurements were performed over a frequency range of 1 MHz to 100 mHz using a 10 mV applied alternating current signal.

1.5 Electrochemical cell assembly and analysis

The two electrodes coin cells (2032-type) were assembled with Li_4-Ph-CH_3P and Li_4-Ph-PhP as working electrode material, and lithium foil as counter and pseudo-reference electrode. Glass microfiber filters (Whatman GF/D, Aldrich) were used as separators. A typical working electrode was composed of 50 wt.% active material, 40 wt.% of super P carbon and 10 wt.% of PTFE by hand grinding. Mass loading for the positive electrodes was in the range of 3-6 mg cm^{-2}. 2M $LiCF_3SO_3$ and 2M $NaCF_3SO_3$ in diglyme were used as electrolyte for lithium and sodium ion batteries.

1.6 Density Functional Theory Simulation

All calculations were carried out with the Gaussian 16 software.[6] The B3LYP functional was adopted for all calculations. For geometry optimization and frequency calculations, the def2-SVP[7] basis set was used, and the optimal geometry for each compound was determined. The singlet point energy calculations were performed with a larger basis set def2-TZVP basis set. The DFT-D3 dispersion correction with BJ-damping [8] was applied to correct the weak interaction to improve the calculation accuracy. Orbital energy level analysis and density-of-states analysis were performed by Multiwfn package.[9] The visualization of the orbitals for Li_4-Ph-CH_3P, Na_4-Ph-CH_3P, Li_4-Ph-PhP and Na_4-Ph-PhP were achieved using VMD software.

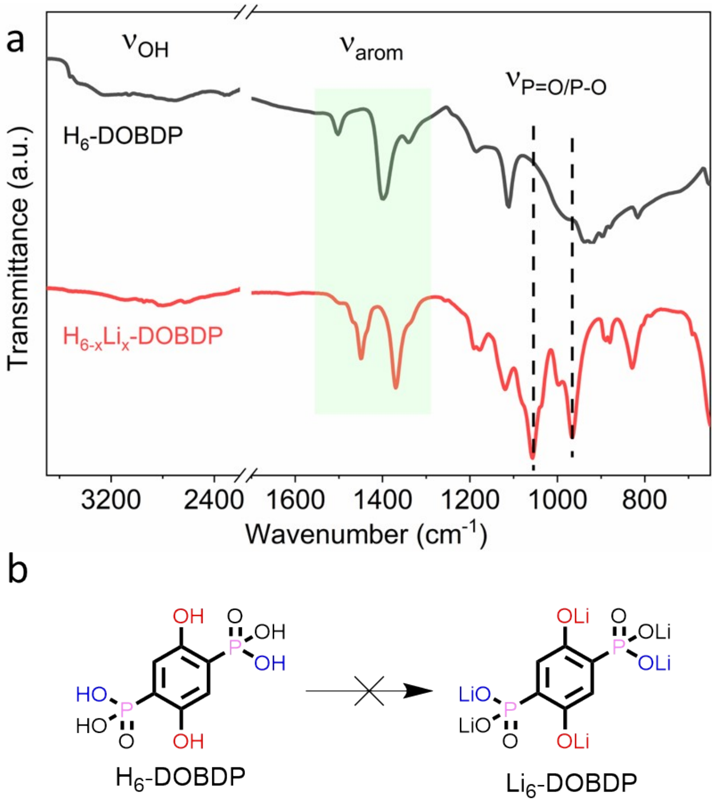

Figure S1. (a) Comparative FTIR analysis of H_6-DOBDP and $H_{6-x}Li_x$-DOBDP, (b) Schematic illustration of lithiation reaction of H_6-DOBDP by conventional liquid acid-base reaction conditions.

Table S1. List of the studied compounds with the full names and abbreviations.

Compounds	Full names	Abbreviations
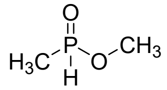	Methyl methyl-phosphinate	CH_3O-CH_3P
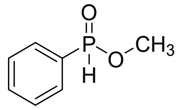	Methyl phenyl-phosphinate	CH_3O-PhP
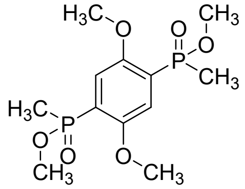	Dimethyl (2,5-dimethoxy-1,4-phenylene) bis(methyl-phosphinate)	$(CH_3O)_4$-Ph-CH_3P
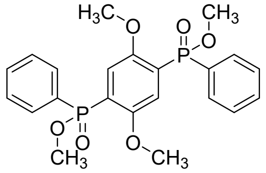	Dimethyl (2,5-dimethoxy-1,4-phenylene) bis(phenyl-phosphinate)	$(CH_3O)_4$-Ph-PhP
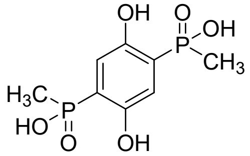	phenylene-1,4-bis(methylphosphinic acid)	H_4-Ph-CH_3P
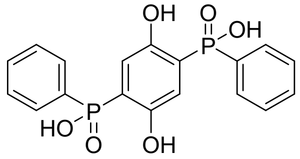	phenylene-1,4-bis(phenylphosphinic acid)	H_4-Ph-PhP
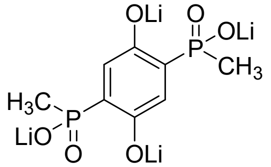	Tetralithium bis(methylphosphinate)	Li_4-Ph-CH_3P
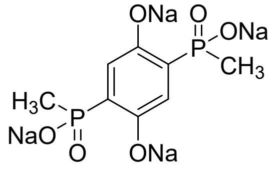	Tetrasodium bis(methylphosphinate)	Na_4-Ph-CH_3P
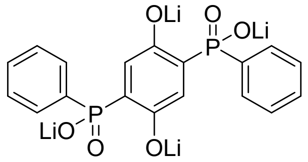	Tetralithium bis(phenylphosphinate)	Li_4-Ph-PhP
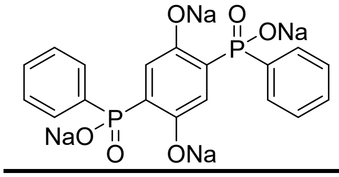	Tetrasodium bis(phenylphosphinate)	Na_4-Ph-PhP

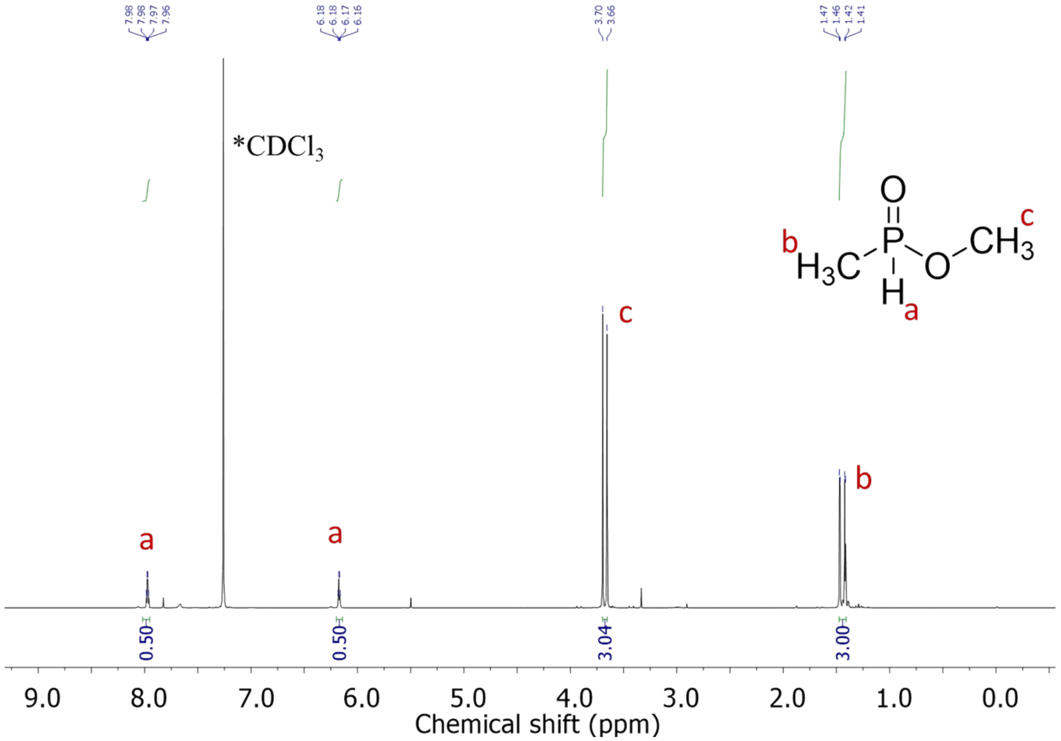

Figure S2. ^{1}H NMR of CH_3O-CH_3P in $CDCl_3$.

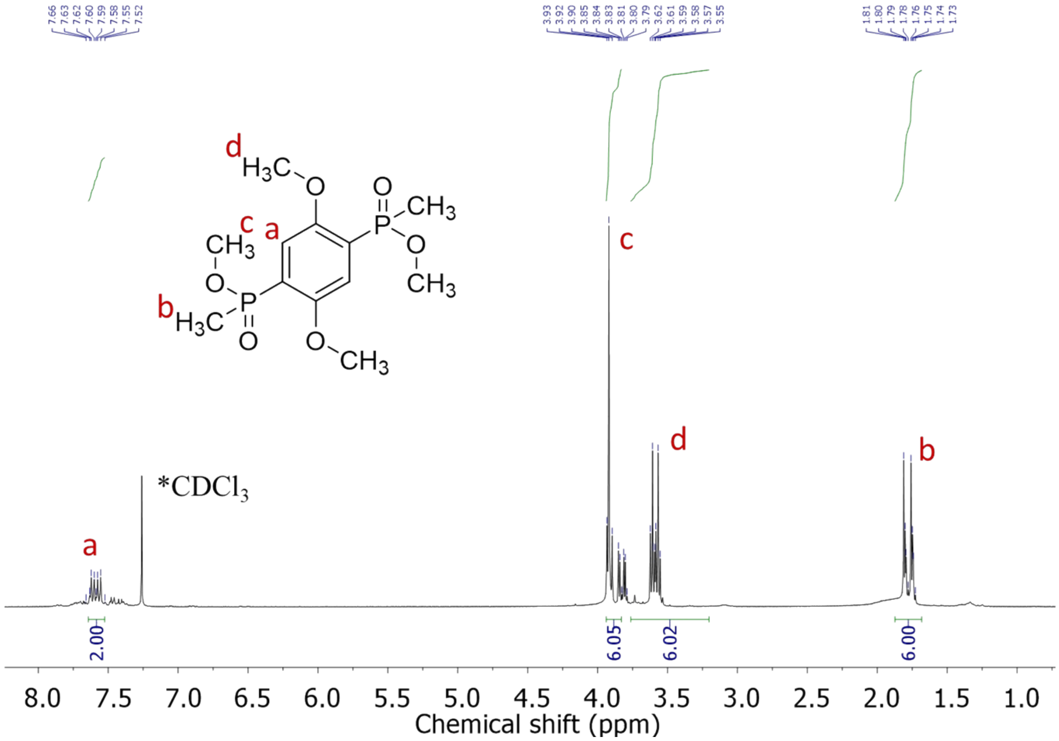

Figure S3. ^{1}H NMR of $(CH_3O)_4$-Ph-CH_3P in $CDCl_3$.

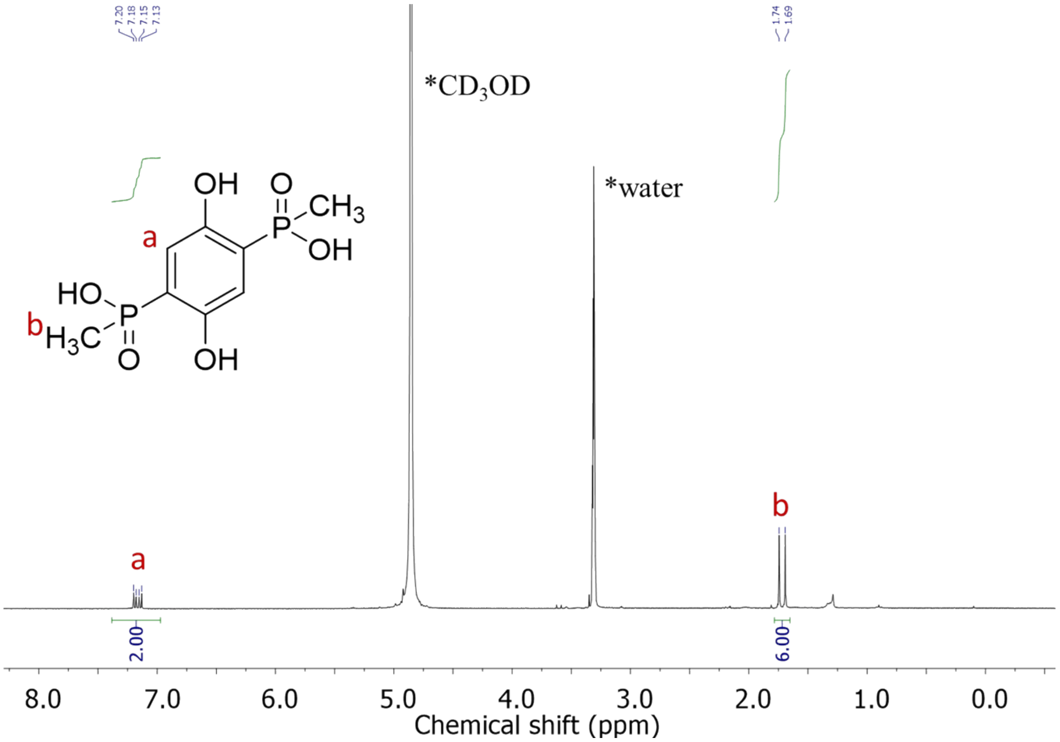

Figure S4. ^{1}H NMR of H_4-Ph-CH_3P in CD_3OD.

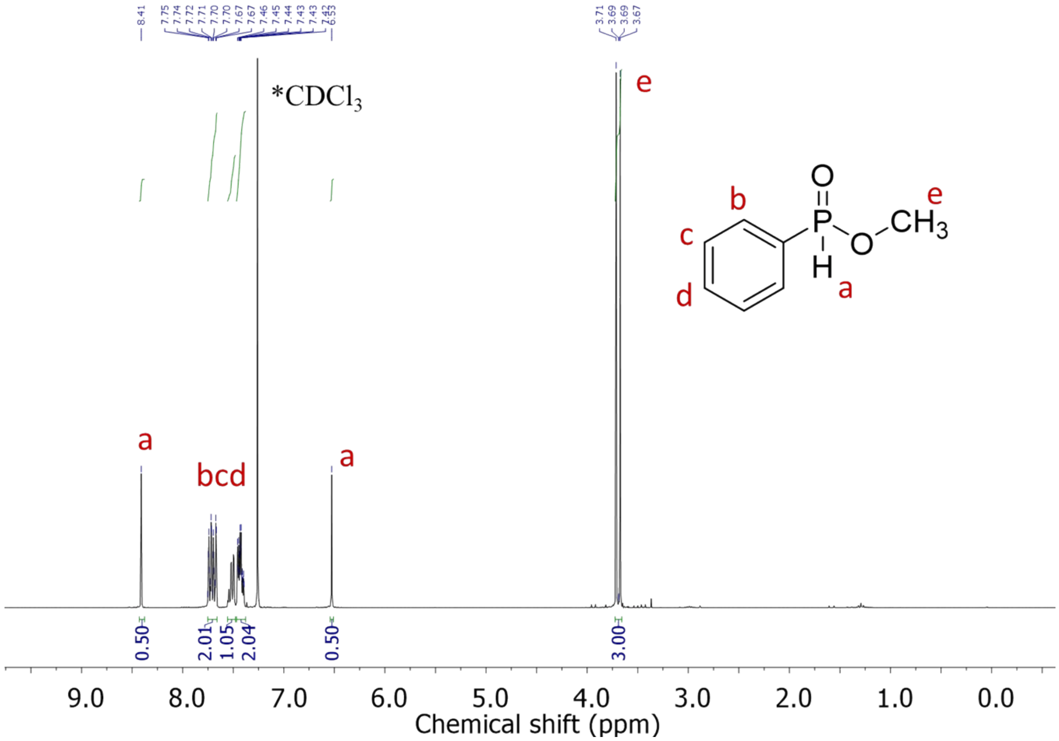

Figure S5. ^{1}H NMR of CH_3O-PhP in $CDCl_3$.

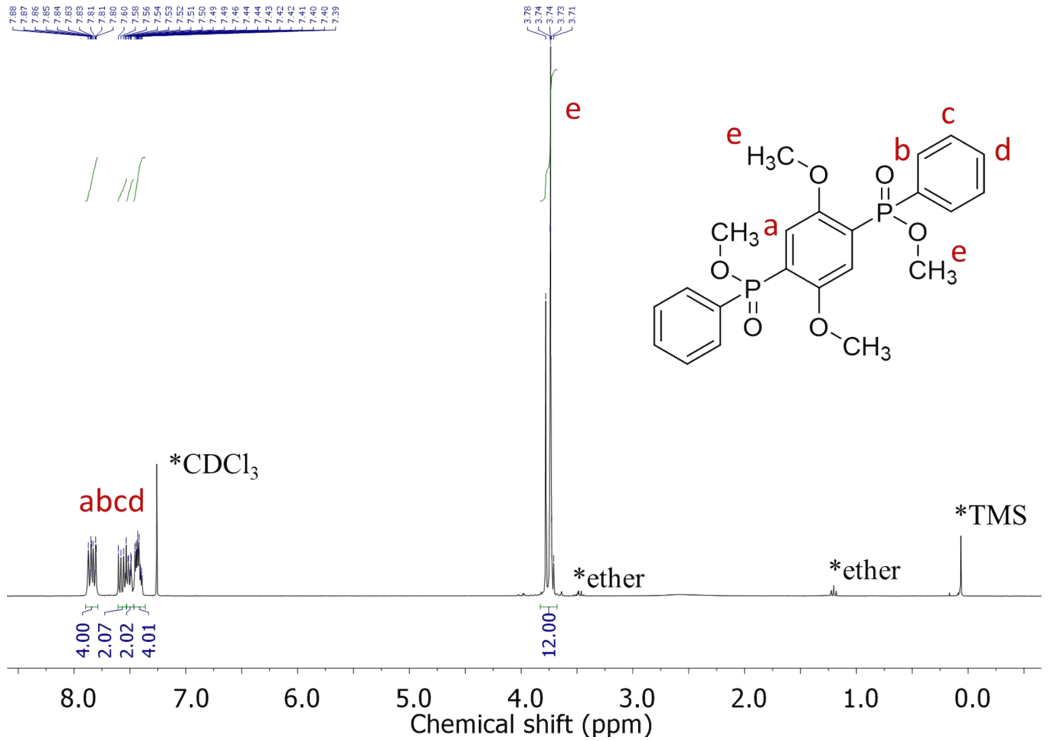

Figure S6. ^{1}H NMR of $(CH_3O)_4$-Ph-PhP in $CDCl_3$.

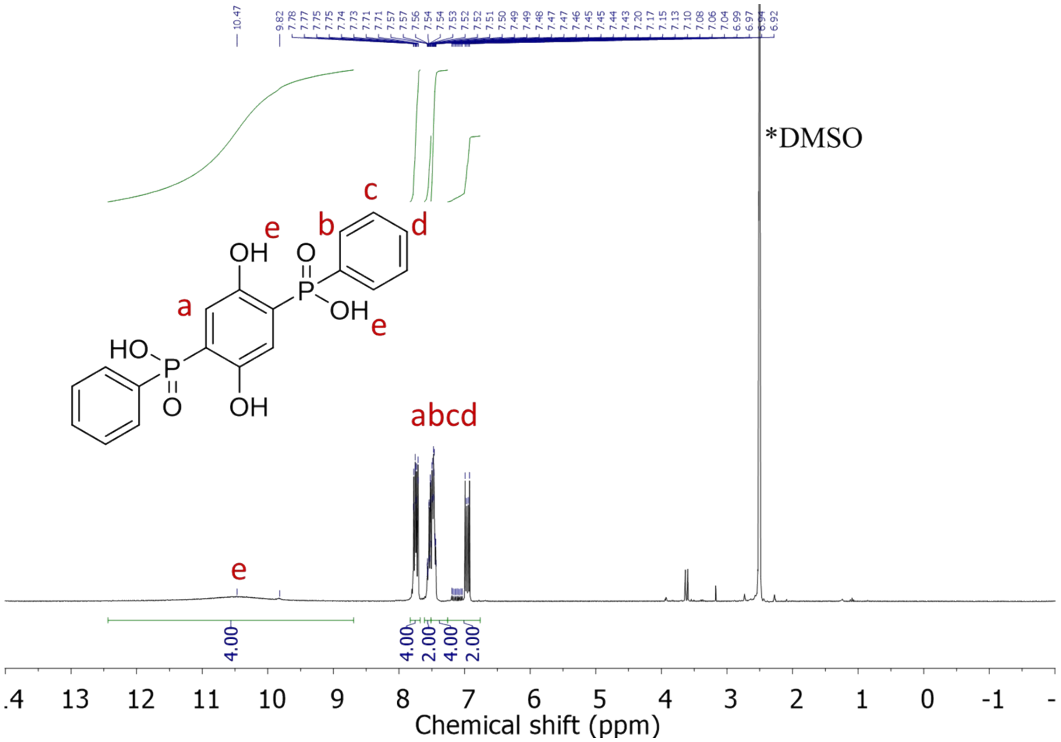

Figure S7. ^{1}H NMR of H_4-Ph-PhP in DMSO-d_6.

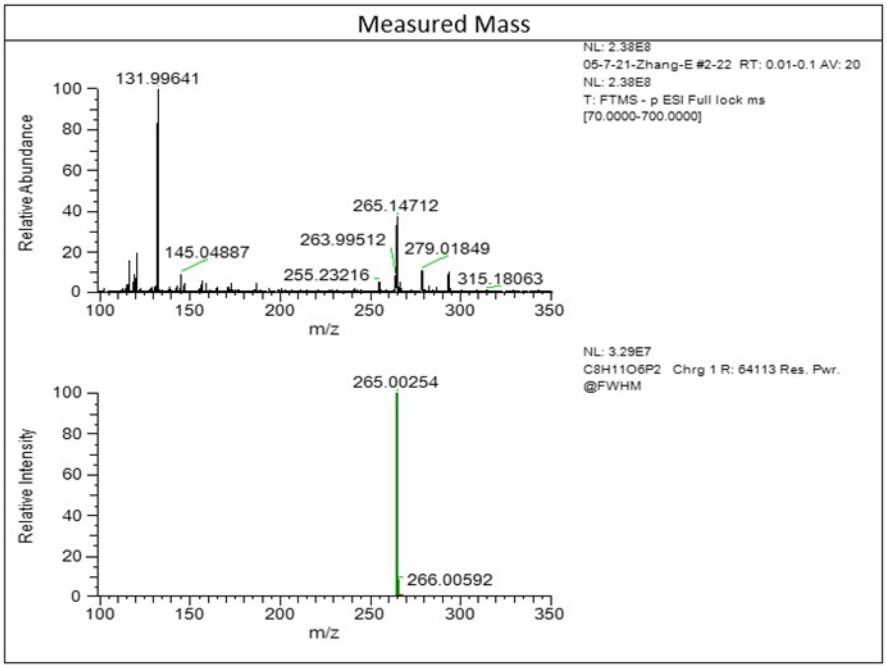

Figure S8. HR-MS of H_4-Ph-CH_3P.

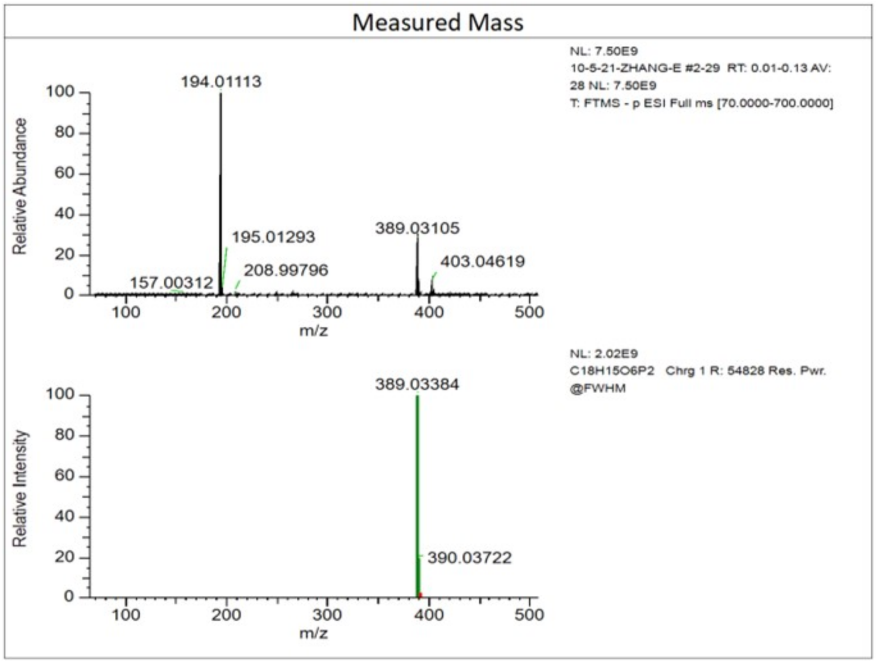

Figure S9. HR-MS of H_4-Ph-PhP.

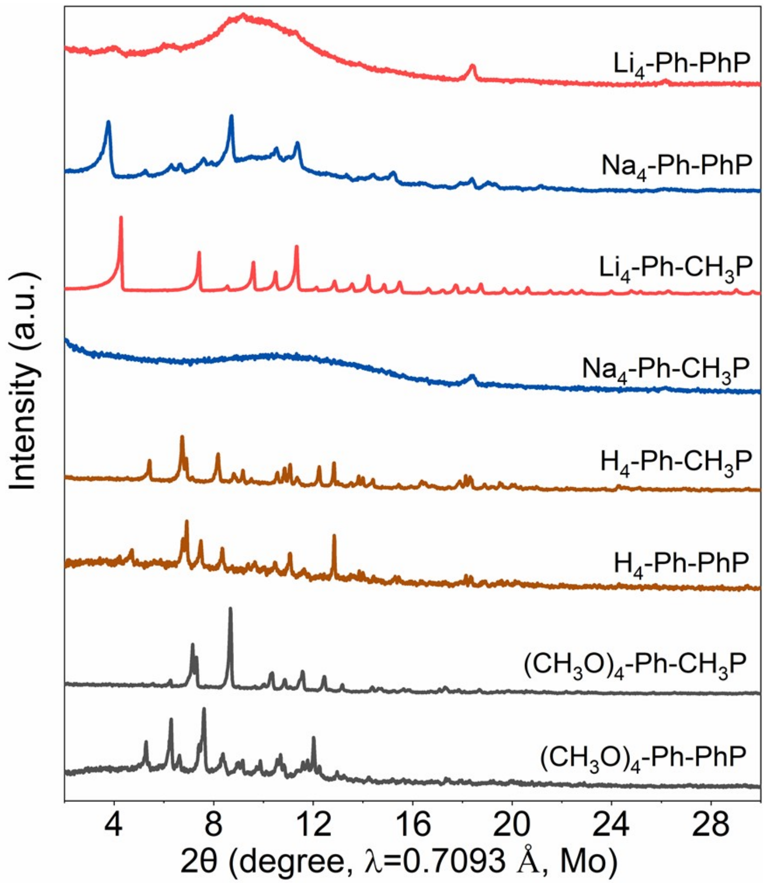

Figure S10. PXRD patterns of synthesized materials.

PXRD analysis revealed a crystalline phase for Na_4-Ph-PhP and Li_4-Ph-CH_3P, and amorphous phases for Li_4-Ph-PhP and Na_4-Ph-CH_3P (**Figure S10**). Furthermore, the diffraction peaks located at 2θ=3.7°, 6.1°, 8.7°, 11.2° and 18.4° suggest the possible similar crystal structure of Li_4-Ph-PhP and Na_4-Ph-PhP.

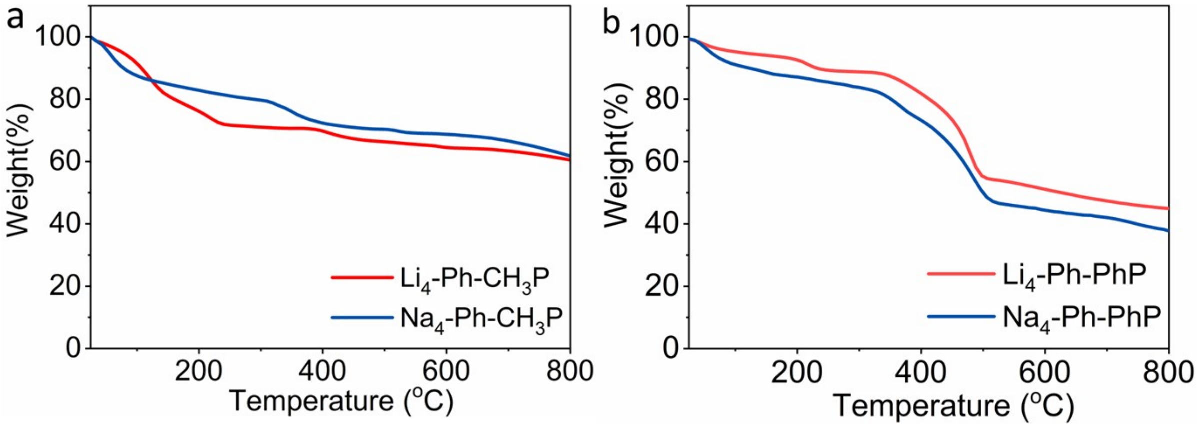

Figure S11. TG of (a) A_4-Ph-CH_3P, and (b) A_4-Ph-PhP, A=Li, Na.

As shown in **Figure S11a**, Li_4-Ph-CH_3P and Na_4-Ph-CH_3P exhibits a weight loss of approximately 30 % and 20 % from room temperature to 260 °C, mainly due to the evaporation of residual methanol solvent. After solvent removal, the thermal stability of Li_4-Ph-CH_3P and Na_4-Ph-CH_3P is around 360 °C and 310 °C, respectively. Li_4-phle-phP and Na_4-phle-phP show noticeable weight loss from the onset of heating to 300 °C, with weight losses of 11 % and 17 % of the initial sample weight, respectively. This is primarily attributed to the slow evaporation of residual methanol solvent from the materials. As the temperature continues to rise from 340 °C to 800 °C, both Li_4-phle-phP and Na_4-phle-phP materials experience weight losses of 43 % and 45 %, respectively, indicating decomposition and carbonization of the materials (**Figure S11b**).

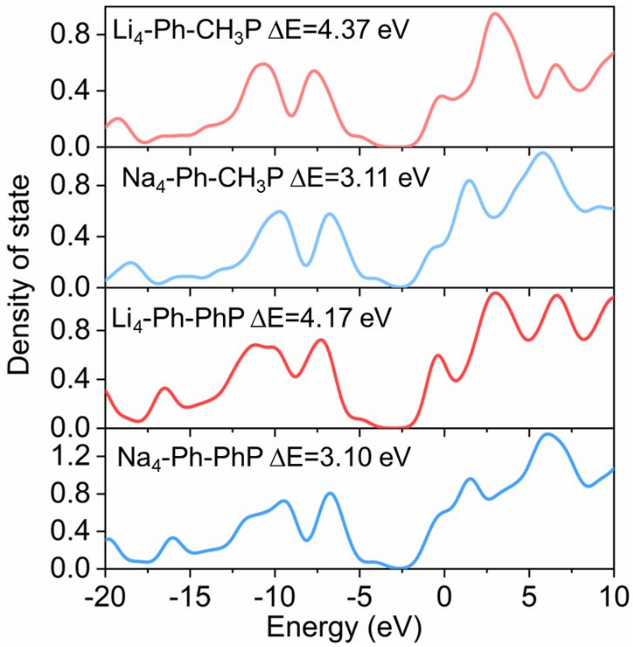

Figure S12. Density of state (DOS) of Li_4-Ph-CH_3P, Na_4-Ph-CH_3P, Li_4-Ph-PhP, and Na_4-Ph-PhP.

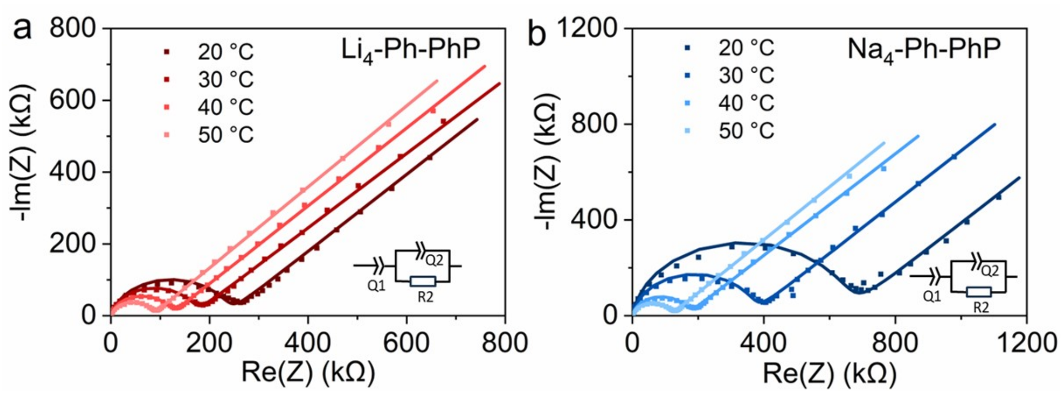

Figure S13. Nyquist plots of (a) Li_4-Ph-PhP and (b) Na_4-Ph-PhP from 20 ℃ to 50 ℃.

Table S2. Ionic conductivity calculation details for Li_4-Ph-PhP and Na_4-Ph-PhP, pellet area A=0.785 cm ^{2}, L is the thickness of the pellets.

Sample	T (℃)	R_2 (KΩ)	L (cm)	σ (S cm^{-1})
Li_4-Ph-PhP	50	82.365	0.040	6.1×10^{-7}
	40	118.153	0.036	3.8×10^{-7}
	30	163.575	0.034	2.6×10^{-7}
	20	232.779	0.030	1.6×10^{-7}
Na_4-Ph-PhP	50	113.903	0.040	4.4×10^{-7}
	40	162.846	0.036	2.8×10^{-7}
	30	357.652	0.040	1.4×10^{-7}
	20	638.128	0.042	8.3×10^{-8}

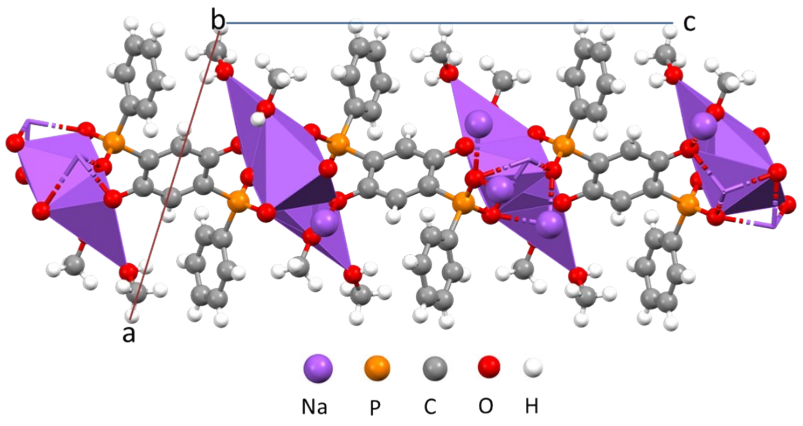

Figure S14. X-ray crystal structure of Na_4-Ph-PhP·CH_3OH.

Table S3. Crystal data and structure refinement for Na_4-Ph-PhP.

Empirical formula	$C_{22} H_{28} Na_4 O_{10} P_2$
Formula weight	606.34
Temperature/K	150.15
Crystal system	monoclinic
Space group	P 21/c
a/Å	12.6672(14)
b/Å	6.1624(6)
c/Å	18.016(2)
α/°	90
β/°	106.967(12)
γ/°	90
Volume/Å^3	1345.1(3)
Z	2
ρ_{calc} g/mm^3	1.497
μ /mm^{-1}	0.814
F(000)	628
Crystal size/mm^3	0.25 × 0.03 × 0.01
Index ranges	-14 ≤ h ≤ 14, -6 ≤ k ≤ 6, -20 ≤ l ≤ 19
Reflections collected	7054
Independent reflections	1878[R(int) = 0.1125]
Data/restraints/parameters	1878/0/127
Goodness-of-fit on F^2	1.116
Final R indexes [I>=2σ (I)]	R_1 = 0.0878, wR_2 = 0.1694
Final R indexes [all data]	R_1 = 0.1318, wR_2 = 0.1060

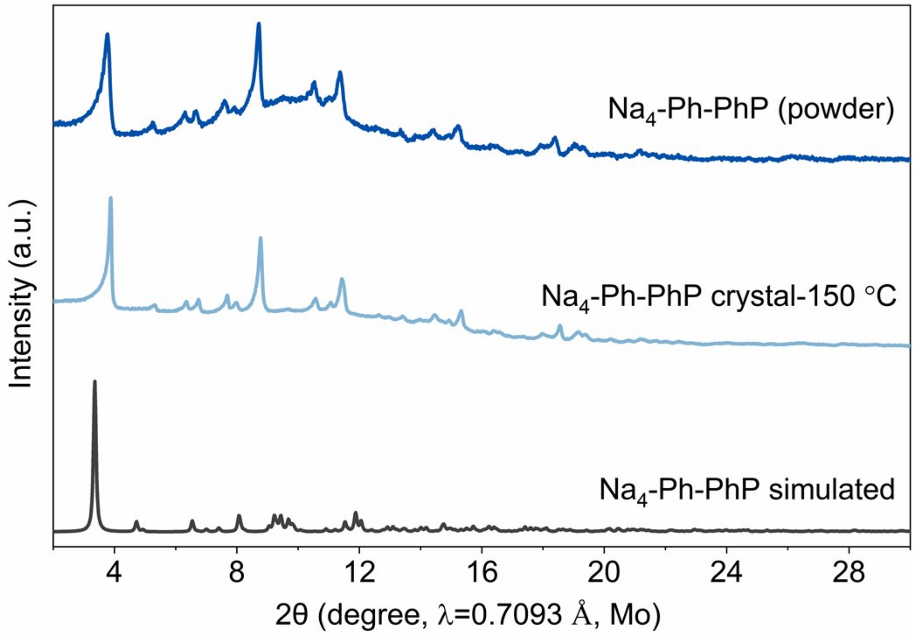

Figure S15. PXRD patterns of Na_4-Ph-PhP materials.

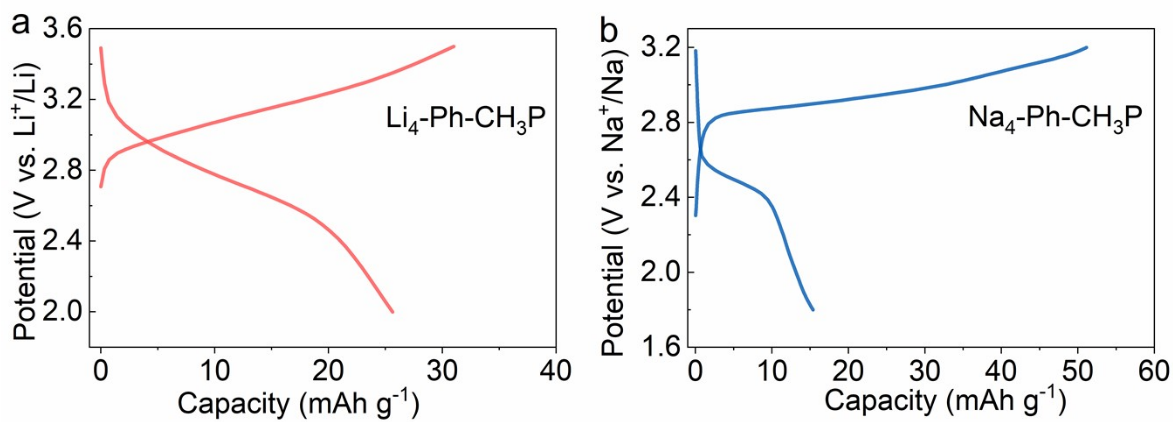

Figure S16. The first charge and discharge voltage profiles at a rate of 0.1C for (a) Li_4-Ph-CH_3P, (b) Na_4-Ph-CH_3P electrode materials.

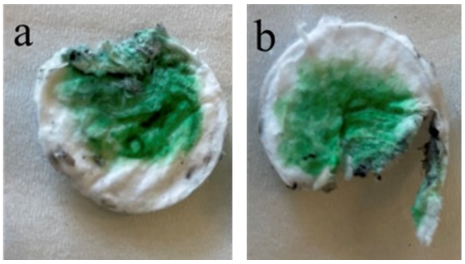

Figure S17. Photographs of glass fiber separator from (a) Li_4-Ph-CH_3P and (b) Na_4-Ph-CH_3P cells after the first charge/discharge process.

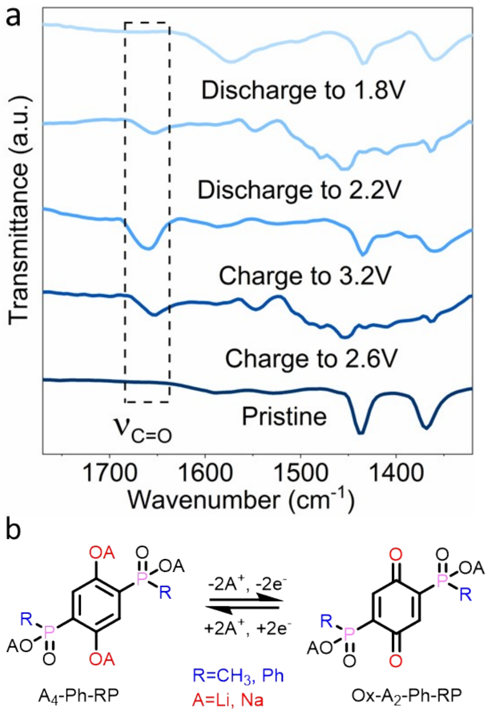

Figure S18 (a) Ex-situ FTIR spectroscopy analysis of Na_4-Ph-PhP in different charge and discharge state, (b) redox mechanism of A_4-Ph-RP.

To have a better comprehension of the redox mechanism for Na_4-Ph-PhP electrodes, ex-situ FTIR analysis was performed over one full charge/discharge cycle (**Figure S18**). The results reveal the appearance of a new band at ~1654 cm^{-1} (charge to 2.6 V) due to the formation of quinonoid/semiquinone radical (1e$^-$ oxidized), which became more intense and shifted to high frequency indicating the formation quinone band (C=O, 2e$^-$ oxidized) when fully charged to 3.2 V. During the discharging process, the reversible appearance of (1e$^-$ reduced) semiquinone radical C=O vibration bond has partially faded (discharge to 2.2 V) and then completely vanished (1.8 V, phenolate, 2e$^-$ reduced), indicating the reversibility of the electrochemical process at the molecular level.

References

[1] CrysAlisPro, version 1.171.37.35; Rigaku Oxford Diffraction.
[2] Sheldrick, G. M. (2015). Acta Cryst. A71, 3-8.
[3] Sheldrick, G. M. (2015). Acta Cryst. C71, 3-8.
[4] Spek, A. L. (2009). Acta Cryst. D65, 148-155.
[5] C. F. Macrae, I. Sovago, S. J. Cottrell, P. T. A. Galek, P. McCabe, E. Pidcock, M. Platings, G. P. Shields, J. S. Stevens, M. Towler and P. A. Wood, J. Appl. Cryst., 53, 226-235, 2020.
[6] M. J. Frisch, G. W. Trucks, H. B. Schlegel, G. E. Scuseria, M. A. Robb, J. R. Cheeseman, G. Scalmani, V. Barone, G. A. Petersson, H. Nakatsuji, X. Li, M. Caricato, A. V. Marenich, J. Bloino, B. G. Janesko, R. Gomperts, B. Mennucci, H. P. Hratchian, J. V. Ortiz, A. F. Izmaylov, J. L. Sonnenberg, Williams, F. Ding, F. Lipparini, F. Egidi, J. Goings, B. Peng, A. Petrone, T. Henderson, D. Ranasinghe, V. G. Zakrzewski, J. Gao, N. Rega, G. Zheng, W. Liang, M. Hada, M. Ehara, K. Toyota, R. Fukuda, J. Hasegawa, M. Ishida, T. Nakajima, Y. Honda, O. Kitao, H. Nakai, T. Vreven, K. Throssell, J. A. Montgomery Jr., J. E. Peralta, F. Ogliaro, M. J. Bearpark, J. J. Heyd, E. N. Brothers, K. N. Kudin, V. N. Staroverov, T. A. Keith, R. Kobayashi, J. Normand, K. Raghavachari, A. P. Rendell, J. C. Burant, S. S. Iyengar, J. Tomasi, M. Cossi, J. M. Millam, M. Klene, C. Adamo, R. Cammi, J. W. Ochterski, R. L. Martin, K. Morokuma, O. Farkas, J. B. Foresman, D. J. Fox, Wallingford, CT 2016.
[7] a) F. Weigend, R. Ahlrichs, Phys Chem Chem Phys 2005, 7, 3297; b) F. Weigend, Phys Chem Chem Phys 2006, 8, 1057.
[8] S. Grimme, S. Ehrlich, L. Goerigk, J Comput Chem 2011, 32, 1456.
[9] T. Lu, F. Chen, J Comput Chem 2012, 33, 580.
